# To Share or Not to Share: Malaysian Healthcare Professionals' Views on Localized Prostate Cancer Treatment Decision Making Roles

**DOI:** 10.1371/journal.pone.0142812

**Published:** 2015-11-11

**Authors:** Yew Kong Lee, Ping Yein Lee, Ai Theng Cheong, Chirk Jenn Ng, Khatijah Lim Abdullah, Teng Aik Ong, Azad Hassan Abdul Razack

**Affiliations:** 1 Department of Primary Care Medicine, Faculty of Medicine, University of Malaya, 50603 Kuala Lumpur, Malaysia; 2 Department of Family Medicine, Universiti Putra Malaysia, 43400 Serdang, Selangor, Malaysia; 3 Department of Nursing Science, Faculty of Medicine, University of Malaya, 50603 Kuala Lumpur, Malaysia; 4 Department of Surgery, Faculty of Medicine, University of Malaya, 50603 Kuala Lumpur, Malaysia; University of Stirling, UNITED KINGDOM

## Abstract

**Aim:**

To explore the views of Malaysian healthcare professionals (HCPs) on stakeholders’ decision making roles in localized prostate cancer (PCa) treatment.

**Methods:**

Qualitative interviews and focus groups were conducted with HCPs treating PCa. Data was analysed using a thematic approach. Four in-depth interviews and three focus group discussions were conducted between December 2012 and March 2013 using a topic guide. Interviews were audio-recorded, transcribed verbatim, and analysed thematically.

**Findings:**

The participants comprised private urologists (n = 4), government urologists (n = 6), urology trainees (n = 6), government policy maker (n = 1) and oncologists (n = 3). HCP perceptions of the roles of the three parties involved (HCPs, patients, family) included: HCP as the main decision maker, HCP as a guide to patients’ decision making, HCP as a facilitator to family involvement, patients as main decision maker and patient prefers HCP to decide. HCPs preferred to share the decision with patients due to equipoise between prostate treatment options. Family culture was important as family members often decided on the patient’s treatment due to Malaysia’s close-knit family culture.

**Conclusions:**

A range of decision making roles were reported by HCPs. It is thus important that stakeholder roles are clarified during PCa treatment decisions. HCPs need to cultivate an awareness of sociocultural norms and family dynamics when supporting non-Western patients in making decisions about PCa.

## Introduction

Decision making about early prostate cancer is a complex, preference-sensitive choice involving many different options [1, 2]. Almost fifty percent of patients with prostate cancer find the decision difficult and distressing [3]. Upon diagnosis, most patients prefer to actively collaborate with their healthcare providers to make a decision [4]. However, a discrepancy often exists between patients’ actual and preferred decision making roles in cancer treatment [5, 6].

Besides healthcare providers, patients with prostate cancer also wish to involve their families and partners in the decision. In one study, all patients with prostate cancer preferred a collaborative decision role with their partners [7]. Family support helps patients with information-gathering, active decision-making and clarifying a patient’s quality-of-life preferences [8]. Family roles are especially relevant in the Malaysian context where family members have been reported to play active roles in various medication and surgical choices [9–11].

Given the good survival rates for prostate cancer and the importance of quality-of-life issues stemming from treatment side effects, shared informed decision making is increasingly advocated as the ideal model for prostate cancer treatment decisions [8]. Decision aids have been developed to facilitate shared decision making in prostate cancer; these have been shown to improve patient knowledge, reduced decisional distress and improved decisional satisfaction[12].

It is generally perceived that patients from non-Western, low-and middle-income countries experience lower levels of involvement in decision making and that paternalistic consultation styles are prevalent [13]. For example, a situational analysis in Malaysia reported that patients were not involved in decision making during clinical consultations [14]. However, patient involvement varies across Asia; a study by Lam et al reported that 73% of Chinese women reported having been offered a choice regarding breast cancer treatment [15]. Reasons for this variation are little understood; a systematic review on shared decision making in non-Western cultures found no studies describing barriers or facilitators to patient participation or physician attitudes toward patient involvement [13].

In Malaysia, prostate cancer incidence is low with a population age-adjusted incidence rate of 6.2 per 100,000, compared to the United States at 160 per 100,000 [16, 17]. Hence, the experience of the healthcare professionals and the resources available in guiding patients through the decision making process is limited in Malaysia compared to the Western world. Understanding how healthcare professionals from these settings view decision making roles will help to shed light on factors underlying discrepancies between actual and preferred roles. Therefore, this study aimed to explore the views of healthcare professionals (HCPs) on the roles of patients, families and HCPs in treatment decision making of early prostate cancer in Malaysia.

## Methods

### Design

A qualitative methodology was used due to the exploratory nature of the research [18–20]. For this purpose, individual in-depth interviews and focus group discussions using a semi-structured topic guide were conducted. This study was part of a larger study that aimed to develop a patient decision aid for supporting patients with early prostate cancer in treatment decision making.

### Setting

The study was conducted in Malaysia which is a middle income developing country with a multi-ethnic society. Patients here are free to choose between government-subsidised public health services or private sector, which is fee-for-service. Most patients diagnosed with prostate cancer are first seen by an urologist and, if necessary, referred to an oncologist.

This study involved HCPs from both public and private hospitals in the country. HCPs from 8 out of 14 states in Malaysia (Kuala Lumpur, Selangor, Penang, Sabah, Sarawak, Kelantan, Pahang and Johor) were recruited. Key policy makers who were involved in developing and implementing the government national prostate treatment plan were also included.

### Sampling

We used purposive sampling to identify the HCPs who were involved in the treatment of prostate cancer. A ‘snowballing’ technique was used to recruit participants, where HCPs that we had interviewed were asked to identify other HCPs that were involved in prostate cancer treatment. Those identified participated in the subsequent focus group discussions or individual interviews. A total of 22 HCPs were approached and 20 HCPs agreed to participate (response rate 90.9%). Interviews and analyses were done in an iterative manner until no new themes emerged. The recruitment was stopped when the researchers agreed that the analysis had reached thematic saturation.

### Data collection

In-depth interviews and focus groups were conducted with HCPs between December 2012 and March 2013. Four trained researchers (PYL, CJN, KLA, ATC) conducted the interviews using a semi structured topic guide, which was developed based on clinical experience, a conceptual framework (the Ottawa Decision Support Framework) and literature review (Table 1) [21]. The topic guide was used as a template to explore and probe further on issues that emerged during the sessions. The topic guide outline was adapted as results emerged from the data. An assistant took field notes on non-verbal cues and interview dynamics. Focus groups were conducted according to the HCPs’ practice background (three groups: private practitioners, senior consultants in public hospitals, urology residents in public hospitals) to capitalize on shared experiences and ensure homogeneity among the HCPs [22]. Individual in-depth interviews were conducted with policy maker and oncologists because they were unable to attend focus group sessions. Participants were assured of the anonymity and confidentiality of the interview before written consent for interview and audio-recording was obtained.

**Table 1:** Topic guide for in depth interviews and focus group discussions.

*Role and Background*
1. What is your role in caring for men with prostate cancer?
*Managing newly diagnosed patients*
2. Do you face any challenges when managing men with early prostate cancer?
• If yes, what are the challenges you face?
• If no, why not?
• What is the difference between managing men with early and late prostate cancer?
*Patient’s experience*
3. Patients react differently to the diagnosis of prostate cancer. Could you please share with us how your patients react to the diagnosis?
*Patients’ decision making*
4. How does your patient make decisions?
• Timing—when? Describe the follow up process
• Based on information—where do they get the information
• Influence from others—who influences their decision
5. From your experience, who are involved in making decisions about the cancer treatment?
6. What is your role in helping patients make decisions about their treatment?
7. What are the treatment options men with early prostate cancer have?
8. Have you encountered men with early prostate cancer who refuse treatment?
*Helping patients to make decisions*
9. How do you help men with early prostate cancer to make decisions about their treatment?
• What do you do? What do you say?
• Do you explain the benefits of treatment to the patient?
• Do you explain the risks of treatment to the patient?
• Do you explain the disease outcomes with and without treatment?
• Do you discuss the impact of the diagnosis on their work/daily lives/relationships?
• Do you discuss the feasibility of having the treatment, ie. Social support, financial costs, days off work, duration of treatment?
10. Do you refer men with early prostate cancer to other healthcare professionals?
• If yes, who do you refer to?
• What is their role?
• Who are the patients that you refer?
11. What is your view about patients seeking alternative therapy for early prostate cancer?
• Do you discuss alternative therapy with your patients?
• What is your response when they raise the issue of alternative therapy?
• What are some of the alternative therapies patients often use?

The HCPs were informed that the interview focused on patients who had been diagnosed with early prostate cancer. We used open-ended questions and only used prompts if key issues did not emerge spontaneously. The HCPs were asked about the roles of patients, families and HCPs in treatment decision making of patients who were diagnosed with early prostate cancer. Each interview lasted between 60 to 80 minutes. All interviews were audio recorded and transcribed verbatim.

### Data analysis

Thematic analysis was used to analyse the data [23]. Initially, three trained researchers (PYL, CJN, YKL) coded two interviews line-by-line to develop an initial list of nodes. A process of constant comparison was employed, whereby subsequent interviews were coded using this list and new nodes were added to the list upon consultation with the research team.

The nodes were collated into broader categories based on thematic similarities in monthly face-to-face research meetings. All codings were checked by at least two researchers to ensure consistency and a consensus was reached on the final list of nodes. Data collection stopped when data saturation was reached. Nvivo 10 software was used in the data management. Quotes that best captured the essence of the themes were extracted.

The team underwent constant reflection and open discussion throughout the interviews and analysis to reduce possible biases. The researchers involved in the interviews and analysis comprised a nurse, three family physicians, and a psychologist.

### Ethics

Ethics approval was obtained from the Medical Research Ethics Committee, Ministry of Health Malaysia (KKM/NIHSEC/08/0804/P12-735).

## Results

We conducted three focus group discussions and four in-depth interviews. A total of 20 HCPs from 14 institutions participated in the study comprising: private urologists (n = 4), government urologists (n = 6), urology residents (n = 6), government policy maker (n = 1) and oncologists (n = 3). There were 17 male and three female participants. The focus groups comprised urologists in private practice (n = 4), senior urologists from the public sector (n = 6) and urology residents (n = 6).

HCPs identified three parties that were involved in the decision making process: HCPs, patients and family. Two main themes emerged: HCPs’ preferences for their own decisional roles vis-à-vis the patient and their families, and HCPs’ perceptions of patients’ preferences vis-à-vis HCPs and their families.

### Theme 1: HCPs’ decision making role preferences

HCPs views on their own decision making roles in prostate cancer could be divided into three categories: HCP as the main decision maker, HCP as a guide to patients’ decision making, and HCP as a facilitator to family involvement.

#### HCPs make the decision for the patient

Some HCPs considered themselves to be the main decision makers for prostate cancer treatment because they were the experts on medical issues. They would decide which type of treatment was best for the patient. HCPs would try to sway patients towards the option that they believed was best for the patient by priming patients about which option was best for them. While socio-demographic factors were evaluated in deciding which option was best, one HCP relied on an intuition of what the patient wanted ‘deep in his heart’ (HCP 8, urology resident).

I think is the physician that makes the biggest decision…because we’re the guys who can talk to them, who can answer their questions.

HCP 1 (policy maker)

I’ve got one patient who is quite young, so I felt he deserved surgery very much. And then, just be fair, I let him see the oncologist, and then I know from deep in his heart he wants surgery. But he went there and came back he’s okay with radiotherapy. So I talked to him again, and again, finally he decided on surgery. So sometimes patients, they are willing to fall on decisions based on what the doctors are offering them.

HCP 8 (trainee urologist)

#### HCP as a guide to the patients

Some HCPS played the role of a guide when supporting patients in decision making. This included explaining the treatment options or clarifying information that the patient brought to the consultation. As one private urologist (HCP 20) said “(the patients) just want to know more and (so you) explain that to them and then you just guide them you know, the final decision is still up to them”. An awareness of treatment choice equipoise was a key factor for wanting patients to make the final decision.

I never make decision for patients. It’s always patients make the decision for themselves. I’d share the information with them, and uh…you know …advise them…yeah, these are the options, and then…let them make the decision…Yes, because as far as I’m concerned about prostate cancer all three…uh… options are reasonable options. It’s not been shown to be inferior…one compared to the other.

HCP 3 (government oncologist)

Part of this ‘guide’ role involved a self-awareness of maintaining an “independent and neutral” stance towards the various treatment options (HCP 20, private urologist). In practice, this involved maintaining a degree of self-control in what was, or was not, said to the patient in the consultation. HCPs felt they had to withhold their own opinion about which treatment was best. Some HCPs acknowledged that they were most likely to recommend the treatment option that they were familiar with e.g. surgeons would recommend surgery.

I have an idea about what I think is best. I never tell them what I think it is. They make their decision. But if they cannot make the decision, then I’ll tell them, I think this is what the best for you. Like for these reasons.

HCP 18 (private urologist)

I mean being a surgeon of course we are more biased towards giving of what you can, rather than you talking about radiotherapy. But at the end of the day we’ll see whether the disease itself, the patient’s age, whether which is important, and of course we’ve got a role in influencing patient which at the end of the day, still as a consenting decision, shared decision, but of course the surgeon’s got the role in influencing the patient.

HCP 7 (urology resident)

However, it was not easy to maintain this neutral stance with every patient. Although HCPs would allow patients with higher education levels to decide for themselves, they would prime patients with lower education towards the option that they felt was best for them.

It depends on the pool of the patient that we are seeing. It varies. Like in K_, usually I prime the patient which is the best decision that is the best for you. If let’s say I’m seeing a more educated group, I’ll let them decide. At the end they have to decide. I give them percentage on what the things are, the odds are. And then they decide. Whereas at the lower socioeconomic level, they have a bit of disadvantages. Then I’ll try to prime them towards the best choice, which is the best for them. It depends on the group of people you are seeing.

HCP 10 (urology resident)

As one HCP said, the goal of guiding patients was to reach a consensus. This consensual decision was important as forcing the patient to choose what the HCP wanted could lead to regret later on.

I mean my practice is always been should be a consensus, you can’t force it upon the patient what you want to do. We just tell them like everyone else, what’s the options and what are the expected outcomes and because if we tell them that, ‘look, surgery is best’. But in their mind, they do not want surgery. Then you do it. They do it because of what you tell them, they will still not be happy at the end of the day. I mean it has to, because it’s their life. I mean we can facilitate the decision. But I think has to be a consensus what they want and, balance between what they want and what’s the reality.

HCP 5 (trainee urologist)

#### HCPs as facilitators to patient’s family involvement

In some cases, family members, usually the children, made the decision for some older patients. HCPs attributed this involvement to Malaysia’s close-knit family culture.

Decision making I think in our culture is basically family based. So it’s not individually patient, it’s family. If the family is not there then they can’t discuss. And they would not decide on their own as well… They’ll bring their family back or they go back to their family, whichever is convenient.

HCP 10 (urology resident)

Thus, HCPs would actively encourage patients to involve their families in the decision by advising the patient to discuss the decision with their families at home and asking that the family be present at the subsequent consultation.

Usually I don't want them to make decision on the first talk, if they come alone I say that you know, you go back and talk to the family and then probably I’ll schedule another session with the family.

HCP 17 (Private urologist)

The family-based decision making role could be on the wane as one HCP noticed a shift over the years in the decisional role away from the family to the patient himself.

Compared to may be five, ten years ago these days, patients normally make the decision themselves. The family members would be there but it’s quite rare to see management at the end to be decided by family members.

HCP 3 (government oncologist)

### Theme 2: HCPs’ perceptions of patients’ preferred decisional role

Besides their own decisional role preferences, HCPs also described their perceptions on the type of decisional role the patients’ themselves preferred during prostate cancer treatment.

### Patients prefer HCPs to make the decision

Some patients allowed the HCP to make the decision for them; this stance would harmonize with some HCPs’ preference to be the main decision maker. The scenarios described ranged from passive acceptance of the HCP’s treatment recommendation, to an active request for the HCP to decide for them if patients had difficulty understanding the disease.

Some patients, err, when we break out the news, they just accept it and then… after they accept, some just follow whatever we… advise them.

HCP 15 (government urologist)

I would say that the average patient will say, “You decide, doctor”. And the… the problem that we have is that they just don’t understand the disease, they don’t know what they have. And they leave it to our hands, you know.

HCP 14 (government urologist)

#### Patients prefer to make the decision themselves

HCPs said that some patients would make the decision themselves and were decisive on which treatment option they preferred. In order to maintain this conviction on their treatment option, these patients avoided asking too many questions to reduce the possibility of “exposing themselves” to the influence of their HCP.

Very few patients come and say, “Doctor, I cannot think, you think for me…” Very rarely a patient will come and tell me that. They actually make up their minds on their own.

HCP 1 (policy maker, government urologist)

Yeah… patients occasionally ask (the doctor about their opinion), and if they ask I would say, you know. But smart patients would not ask. Smart patients, if they ask, they know that they are exposing themselves to your manipulation already.

HCP 17 (private urologist)

## Discussion

This study sheds light on HCPs’ preferred decisional roles and HCPs’ views on patients’ preferred roles in prostate cancer treatment.

Some of the HCPs in this study went against the grain of stereotypical Asian paternalism. Although literature tends to portray that paternalism is prevalent in Asian clinical consultations [24–26], including Malaysia [14, 27], this study identifies a context in which this is not necessarily always true; what is salient about the nature of prostate cancer treatment is the equipoise between treatment options. Indeed, paternalism may be reduced if more critical awareness of equipoise is developed for a broader variety of medical decisions.

Maintaining the stance of a guide required HCPs to adopt a number of strategies. One strategy was for HCPs to withhold their own opinion about which treatment they thought was best. However, studies in Chinese women with early breast cancer (a disease with similar treatment choices to early stage, localized prostate cancer) report that patients appreciate the recommendation of a surgeon as they lack the background information to make decisions; the doctor’s recommendation serves as a proxy for missing information [28]. Thus, even if HCPs do not share their opinions about which treatment they think is best, they should still share information in an unbiased manner [28]. One implication for practice is the need to develop Malaysian patient decision aids which are adapted to suit the culture, address unique patient concerns, and present unbiased information on locally available options; such tools would help patients get the information they need [29].

Doctors who preferred a more paternalistic role believed that they were knowledgeable about the disease and hence more qualified to make decisions for patients. Doctors would question the patient’s ability to make their own choice if the patient had a lower educational status. Studies elsewhere support the idea that HCPs legitimize paternalism due to a perceived education gap between doctors and patients [30, 31]. However, there is growing evidence from Asian studies that most patients want to be involved in decision making, regardless of their education level [32–34]. Therefore, doctors should not assume that they know what is best for patients, but rather engage and educate patients in order to facilitate an informed decision.

Paternalism may also be due to cultural factors; patients from Asian cultural backgrounds have been reported to feel uneasy about sharing a decision with their doctor [35]. As such, shared decision making can be modified to incorporate a model of participatory decision making which also respects hierarchical Asian culture needs [30]. While maintaining the hierarchy between doctor and patient (e.g. retaining honorific titles in conversation), the doctor may have to consciously and clearly communicate to the patient that shared decision making is the appropriate decision making model for preference-sensitive decisions.

HCPs viewed family members as playing an important role in prostate cancer decisions and sought to involve the family in consultations; this was attributed to the close-knit family culture in Asia. Indeed understanding cultural, religious and traditional values plays an important role in how families and doctors manage illnesses [36]. For example, our study differs from studies elsewhere which report that doctors in Asian cultural settings would conceal information or diagnosis from cancer patients in order to discuss it with the dominant family member; the rationale for this was to preserve family relationships, which were seen to be more important than individual autonomy [27]. It is important for HCPs to be trained in cultural competency in family issues; two important domains are understanding how intimate family relationships are ordered, and understanding the manner in which family crises affect caregiving [36].

A limitation of this study is that it only reports the perspective of HCPs about what they perceive patient’s decision making role to be. This may not be a completely accurate interpretation of patient views; patient perspectives will be explored and reported in the next phase of this study. The strength of the study was the breadth of the sampling frame; we recruited HCPs from all major stakeholder categories, different states and the two main practice settings. Capturing views from diverse perspectives was important as few studies have been conducted on prostate cancer decision making in an Asian context.

### Conclusion

HCPs in Malaysia navigate a range of personal, patient and family roles during prostate cancer treatment decision making. HCPs need to cultivate an awareness of sociocultural norms, family dynamics, personal biases and understanding of a HCP’s role when supporting patients in making the choice. There is a need to develop a culturally-sensitive model of shared decision making which frames the shared decision making process as part of the hierarchical doctor-patient interaction in an Asian clinical context.
